# Internal radiation dosimetry of a ^152^Tb-labeled antibody in tumor-bearing mice

**DOI:** 10.1186/s13550-019-0524-7

**Published:** 2019-06-11

**Authors:** Francesco Cicone, Silvano Gnesin, Thibaut Denoël, Thierry Stora, Nicholas P. van der Meulen, Cristina Müller, Christiaan Vermeulen, Martina Benešová, Ulli Köster, Karl Johnston, Ernesto Amato, Lucrezia Auditore, George Coukos, Michael Stabin, Niklaus Schaefer, David Viertl, John O. Prior

**Affiliations:** 1 Department of Nuclear Medicine and Molecular Imaging, Lausanne University Hospital, Rue du Bugnon 46, CH-1011 Lausanne, CH Switzerland; 2 Institute of Radiation Physics, Lausanne University Hospital, Lausanne, CH Switzerland; 3 ISOLDE/CERN, Geneva, CH Switzerland; 4 Center for Radiopharmaceutical Sciences ETH-PSI-USZ, Paul Scherrer Institute (PSI), Villigen, CH Switzerland; 5 Laboratory of Radiochemistry, Paul Scherrer Institute (PSI), Villigen, CH Switzerland; 6 Institut Laue-Langevin, Grenoble, FR France; 7 Section of Radiological Sciences, Department of Biomedical and Dental Sciences and Morphofunctional Imaging, University of Messina, Messina, IT Italy; 8 Department of Oncology and Ludwig Center for Cancer Research, Lausanne, CH Switzerland; 9 NV5/Dade Moeller, Richland, WA USA

**Keywords:** microPET, Small animal dosimetry, ^152^Tb, OLINDA/EXM® 2.0, Biodistribution, Organ harvesting, TEM-1, Murine phantoms, Spherical model, Radiolabeled monoclonal antibodies

## Abstract

**Background:**

Biodistribution studies based on organ harvesting represent the gold standard pre-clinical technique for dose extrapolations. However, sequential imaging is becoming increasingly popular as it allows the extraction of longitudinal data from single animals, and a direct correlation with deterministic radiation effects. We assessed the feasibility of mouse-specific, microPET-based dosimetry of an antibody fragment labeled with the positron emitter ^152^Tb [(*T*_1/2_ = 17.5 h, Eβ^+^mean = 1140 keV (20.3%)]. Image-based absorbed dose estimates were compared with those obtained from the extrapolation to ^152^Tb of a classical biodistribution experiment using the same antibody fragment labeled with ^111^In.

^152^Tb was produced by proton-induced spallation in a tantalum target, followed by mass separation and cation exchange chromatography. The endosialin-targeting scFv78-Fc fusion protein was conjugated with the chelator *p*-SCN-Bn-CHX-A”-DTPA, followed by labeling with either ^152^Tb or ^111^In. Micro-PET images of four immunodeficient female mice bearing RD-ES tumor xenografts were acquired 4, 24, and 48 h after the i.v. injection of ^152^Tb-CHX-DTPA-scFv78-Fc. After count/activity camera calibration, time-integrated activity coefficients (TIACs) were obtained for the following compartments: heart, lungs, liver, kidneys, intestines, tumor, and whole body, manually segmented on CT. For comparison, radiation dose estimates of ^152^Tb-CHX-DTPA-scFv78-Fc were extrapolated from mice dissected 4, 24, 48, and 96 h after the injection of ^111^In-CHX-DTPA-scFv78-Fc (3–5 mice per group). Imaging-derived and biodistribution-derived organ TIACs were used as input in the 25 g mouse model of OLINDA/EXM® 2.0, after appropriate mass rescaling. Tumor absorbed doses were obtained using the OLINDA2 sphere model. Finally, the relative percent difference (RD%) between absorbed doses obtained from imaging and biodistribution were calculated.

**Results:**

RD% between microPET-based dosimetry and biodistribution-based dose extrapolations were + 12, − 14, and + 17 for the liver, the kidneys, and the tumors, respectively. Compared to biodistribution, the imaging method significantly overestimates the absorbed doses to the heart and the lungs (+ 89 and + 117% dose difference, respectively).

**Conclusions:**

MicroPET-based dosimetry of ^152^Tb is feasible, and the comparison with organ harvesting resulted in acceptable dose discrepancies for body districts that can be segmented on CT. These encouraging results warrant additional validation using radiolabeled biomolecules with a different biodistribution pattern.

## Introduction

The assessment of radiotracer biodistribution in preclinical animal models is an essential part of radiopharmaceutical drug development. The standard method for obtaining biodistribution data has long been the ex vivo measurement of radioactivity concentration in organs of small mammals, dissected at given time points after radiopharmaceutical administration. In addition, there is a growing interest in generating radiation dosimetry data from preclinical studies, in order to better define the radiobiological implications of novel probes, with particular regard to the toxicity and efficacy profiles of therapeutic radiopharmaceuticals [1, 2]. According to the RADAR formalism [3], two terms are required for the calculation of the absorbed dose to a target tissue: the dose factors (DFs) and the time-integrated activity. The DFs incorporate information on the physical properties of the radionuclide, on the geometrical interplay between source and target organs, as well as on their structure. Thanks to the development of realistic small animal geometric phantoms and their implementation in commercial dosimetry software, an accurate calculation of DFs for small animals is now easily accessible [4–6].

The time-integrated radiotracer activity in a given tissue can be estimated either by classical biodistribution studies, or by sequential single-photon (microSPECT) or positron-emission tomographic (microPET) imaging. Imaging techniques are to be preferred in the long term as they allow for longitudinal studies, thereby reducing the number of animals needed for the experiments.

Previous studies have assessed the correlation between in vivo small animal imaging and ex vivo counting for the measurement of activity concentration in single organs using conventional radionuclides such as ^18^F, ^99m^Tc, or ^111^In [7–9]. However, to obtain radiation dose estimates, organ uptake needs to be assessed over time, and organ volumes calculated individually, due to inter- and intra-species heterogeneity. Studies validating mouse-specific, imaging-based dosimetry against radiation dose extrapolations obtained from biodistribution experiments are missing.

The present work was carried out to assess the feasibility of mouse-specific, microPET-based dosimetry of an antibody fragment labeled with the unconventional positron emitter ^152^Tb. Image-based absorbed dose estimates were compared with dosimetry results obtained from the extrapolation to ^152^Tb of a classical biodistribution experiment using the same antibody fragment labeled with ^111^In.

## Methods

### Production of ^152^Tb

^152^Tb is a β^+^ emitter (average β^+^ energy: 1140 keV, 20.3% branching ratio, 17.5 h half-life), which was produced by proton-induced spallation in a tantalum target, followed by an online isotope separation process at ISOLDE (CERN, Geneva, Switzerland) as previously reported [10–12]. Mass 152 ions were implanted into zinc-coated gold foils, and ^152^Tb was then purified at the Paul Scherrer Institute (PSI, Villigen-PSI, Switzerland) using a macroporous strongly acidic cation exchange chromatographic resin (Sykam Vertriebs GmbH, Germany) and eluting the product using α-hydroxyisobutyric acid (α-HIBA; pH 4.7, 0.11 M) [13, 14]. The final product was used directly for labeling purposes.

### Radiolabeling with ^152^Tb and ^111^In

The scFv78-Fc fusion protein targeting endosialin/tumor endothelial marker 1 (TEM1) was obtained as previously described [15] and conjugated with 10 eq. of the chelator *p*-SCN-Bn-CHX-A”-DTPA (Macrocyclics, cat. no. B-355) at 42 °C and pH 9.1 (full details will be reported elsewhere). The final solution was diluted in 0.9% NaCl to give a concentration of 5 mg/mL CHX-DTPA-scFv78-Fc.

A solution of 15 MBq of ^152^Tb in 90 μL α-HIBA was added to 20 μL of CHX-DTPA-scFv78-Fc dissolved in 100 μL of NH_4_OAc buffer (pH 5.4, 0.4 M). After 1 h incubation at 42 °C, ^152^Tb-CHX-DTPA-scFv78-Fc was obtained without further purification with a purity > 95% (iTLC, citrate buffer pH 4.6).

For biodistribution experiments, a commercially available ^111^In chloride solution (200 MBq Mallinckrodt) was added to a mixture of NH_4_OAc buffer (pH 5.4, 0.4 M, 100 μL) and CHX-DTPA-scFv78-Fc (50 μL). After 1 h incubation at 42 °C, an 80–90% conversion was obtained (iTLC, citrate buffer pH 4.6). The product was diluted with 0.9% NaCl and purified by ultrafiltration.

### MicroPET imaging of ^152^Tb-CHX-DTPA-scFv78-Fc

All animal experiments in the present study were conducted according to the Swiss federal law on animal experimentation under the authorization number VD-2993.

TEM-1 expressing Ewing Sarcoma cells (RD-ES, DSMZ, Germany, no. ACC 260) were cultured and grafted subcutaneously in 8–12-weeks-old common gamma KO Balb/c female mice (3 × 10^6^ cells injected in the right flank) weighing about 25 g. Ten-minute images (Energy window 358–664 keV) of 4 RD-ES tumor-bearing mice were acquired on a small animal PET/SPECT/CT device (Albira, Brucker) [16], at three time-points (4, 24, and 48 h) after the intravenous injection of 5–10 MBq ^152^Tb-CHX-DTPA-scFv78-Fc (25–30 μg of total antibody per mouse).

Mice were anesthetized using isoflurane (2% in 1 L/min medical air) and warmed on a heating pad during the scan. Images were reconstructed using a three-dimensional maximum likelihood expectation maximization algorithm with 12 iterations, without any post-reconstruction smoothing. The PET in-plane FOV size was 80 mm with axial extension of 149 mm; reconstructed image voxel size was 0.5 mm isotropic in space. Dead-time, scatter, and random corrections were applied. Co-registered CT (0.2 mA, 35 kV) were used for anatomical localization of uptake and attenuation correction.

### Camera calibration

^152^Tb was quantified and checked for radionuclidic purity using a calibrated N-type high-purity germanium coaxial detector (Eurisys Mesures, Montigny Le Bretonneux, France) in conjunction with a multi-channel analyzer (ORTEC DSPEC Jr., Oak Ridge, TN, USA) and the appropriate spectroscopy software system (Interwinner, Itech Instruments, Rognac, France).

**Fig. 1:**
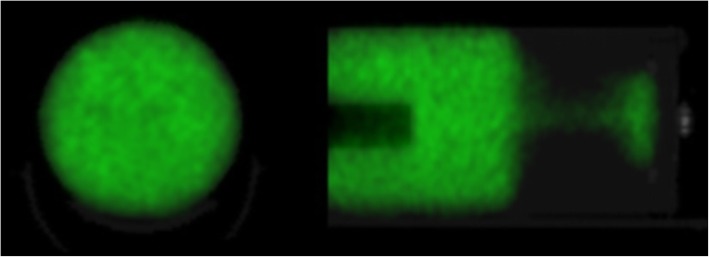
NEMA-NU4 phantom filled with a uniform activity of ^152^Tb, used to derive the count/activity calibration factor

A NEMA-NU4 micro-PET image quality phantom (main volume = 20 mL) was filled with a known activity of 8.2 MBq ^152^Tb in an aqueous solution resulting in an actual activity concentration (A_c,bg_) of 410 kBq/mL (Fig. 1). For phantom imaging and reconstruction, we adopted the same camera settings described above for animal imaging. Quantitative imaging data were obtained by multiplying the measured signal by the scaling factor: SF = (*S*_bg_/*A*_c,bg_)^−1^, where S_bg_ is the average signal measured in the phantom background region.

### MicroPET-based dosimetry

**Fig. 2:**
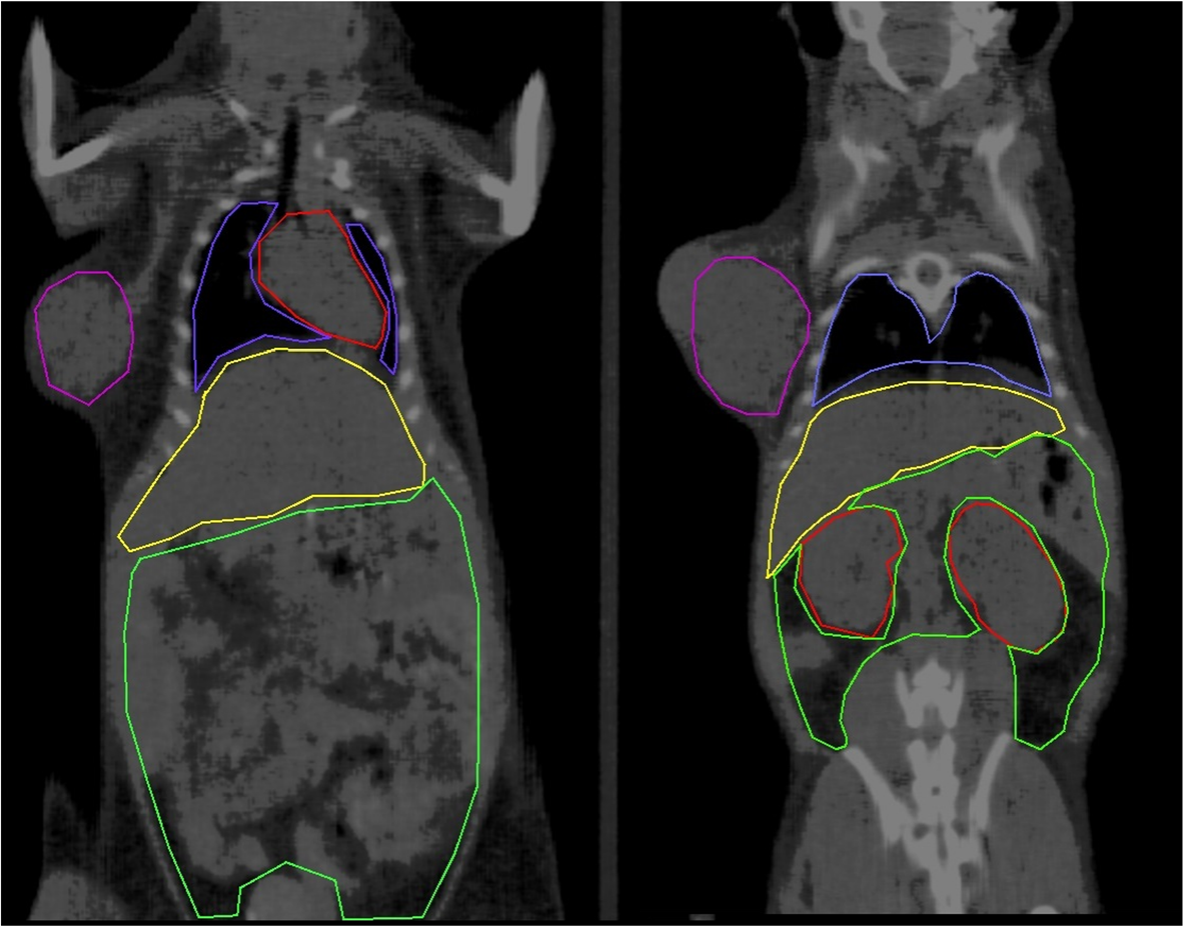
Example of manual CT-based segmentation of most relevant thoracic (heart, lungs) and abdominal organs (liver, kidneys, and intestines), along with a subcutaneous tumor on the right flank

Volumes of interest (VOI) were obtained by manual segmentation of axial CT slices of each micro-PET/CT acquisition using the polygonal segmentation tool available in PMOD (PMOD Technologies, version 3.9, Zurich, Switzerland) for the following regions: heart, lungs, liver, kidneys, intestines, tumor, and whole body (Fig. 2).

For each single animal, the total activity measured in source organs at each time-point was divided by the total administered activity to obtain the normalized time-activity curves (nTAC). nTAC were fitted by a mono-exponential function extended to infinite beyond the last measured data point. Time-integrated activity coefficients (TIACs, in units of MBq·h/MBq) were obtained from analytical integration of the mono-exponential fit for each of the source organs. The TIAC for the rest-of-body was obtained by subtracting the sum of all source organ TIACs from the whole-body TIAC. In order to obtain the organ absorbed doses (mGy/MBq), organ TIACs were used in the input to the OLINDA/EXM® 2.0 (Hermes Medical Solution AB, Stockholm, Sweden, referred as “OLINDA2” thereafter) mouse model (25 g) [6]. For organs that could be resolved by CT, organ volumes were taken from direct segmentation of CT images acquired 24 h post-injection. Organ masses were calculated by multiplying organ volumes by the standard tissue densities indicated in the ICRP110 for humans [17] and used in the input to OLINDA2. This was made possible thanks to a specific mass-rescaling tool in order to replace the standard organ masses available for the mouse model. To estimate the absorbed dose to the large intestine and the small intestine respectively, the TIAC and the mass calculated by segmentation of the whole intestine were partitioned proportionally to the masses of the two single components available in the murine phantom 25 g (i.e., 75% small intestine, 25% large intestine). The absorbed dose to the tumor was obtained using the sphere model available in OLINDA2. The final radiation absorbed doses to organs and tumors, along with their standard deviations, were obtained by averaging the absorbed doses values estimated in the four animals.

Results of the OLINDA2 sphere model were compared with those of an analytical model for determining absorbed fractions and doses in ellipsoids filled with a uniform activity concentration [18–20]. To implement the analytical model, we used the full ^152^Tb emission spectrum taken from [21], which includes the β^+^ spectrum extending up to 2.97 MeV, and all monoenergetic electrons and photons.

The comparison between OLINDA2 and the analytical model for ellipsoidal shapes was carried out for 8 spheres of masses ranging from 0.1 to 10 g. The comparison assumed a TIAC of 1 h and unit-density soft tissue. The relative percent differences between doses calculated with OLINDA2 and with the ellipsoidal model were obtained as

$$ \varepsilon =100\cdot \frac{D_{\mathrm{OLINDA}2}-{D}_{\mathrm{ellipsoid}}}{D_{\mathrm{ellipsoid}}} $$

### Biodistribution studies

Biodistribution of ^111^In-CHX-DTPA-scFv78-Fc was also obtained in 8–12-week-old RD-ES tumor-bearing female common gamma KO Balb/c mice. Mice were injected intravenously with 150 kBq (range 30–440 kBq) ^111^In-CHX-DTPA-scFv78-Fc and sacrificed 4 h (*n* = 3 mice), 24 h (*n* = 5 mice), 48 h (*n* = 4 mice), and 96 h (*n* = 3 mice) post-injection. Before injection, 25 μg of unlabeled scFv78-Fc were added to a saline solution containing 0.2 μg of ^111^In-CHX-DTPA-scFv78-Fc, in order to administer the same amount of total antibody used for microPET studies. Mice were bled by cardiac puncture and sacrificed by cervical dislocation under anesthesia with isoflurane (2% in 1 L/min medical air). Organs were excised and weighed, and radioactivity was counted on a gamma counter (Wallac Wizard, Perkin Elmer, Waltham, MS, USA). For each time point, the activity of each source organ of each single animal was normalized by the total injected activity to obtain nA. For each source organ at each time point, an average nA value was obtained ±1SD.

### Biodistribution-based dosimetry

To allow the comparison with microPET-based dosimetry results, radiation dose estimates of ^152^Tb-CHX-DTPA-scFv78-Fc were extrapolated from ^111^In-CHX-DTPA-scFv78-Fc biodistribution data.

nA values for ^152^Tb were extrapolated from ^111^In measured data points by the application of a scale factor (SF):

$$ SF\left({t}_m\right)=\mathit{\exp}\left[\left(- In(2)/{T}_{p, Tb-152}\right)\times {t}_m\right]/\mathit{\exp}\left[\left(- In(2)/{T}_{p, In-111}\right)\times {t}_m\right] $$

Where *t*_m_ indicates the measured time points (4, 24, 48, and 96 h post-injection, respectively), therefore,

$$ {nA}_{Tb-152}\left({t}_m\right)={nA}_{In-111}\left({t}_m\right)\times \mathrm{SF}\left({t}_m\right) $$

This rescaling procedure compensates for the different physical half-life of the two radioisotopes, assuming the same biological half-life.

The nTAC obtained from all source organs was found to be monotonically decreasing; therefore, to derive TIACs, three analytical mono-exponential fits were applied to the nTACs generated from the average nA, average nA + 1SD, and average nA–1SD data points, respectively.

Consistently with the image-based dosimetric methodology, ^152^Tb-CHX-DTPA-scFv78-Fc organ absorbed doses and tumor doses were obtained using the murine (25 g) model and the sphere model available in OLINDA2, respectively [6]. The standard organ masses of the OLINDA2 mouse model 25 g were replaced by the average organ masses measured across all animals used for the biodistribution experiments. The relative percent difference between absorbed doses obtained from microPET imaging (AD_im_) and the absorbed doses extrapolated from biodistribution (AD_biod_) was calculated as follows:

$$ \mathrm{RD}\left(\%\right)=\left[\left({\mathrm{AD}}_{\mathrm{im}}-{\mathrm{AD}}_{\mathrm{biod}}\right)/{\mathrm{AD}}_{\mathrm{biod}}\operatorname{}\right]\times 100 $$

## Results

**Fig. 3:**
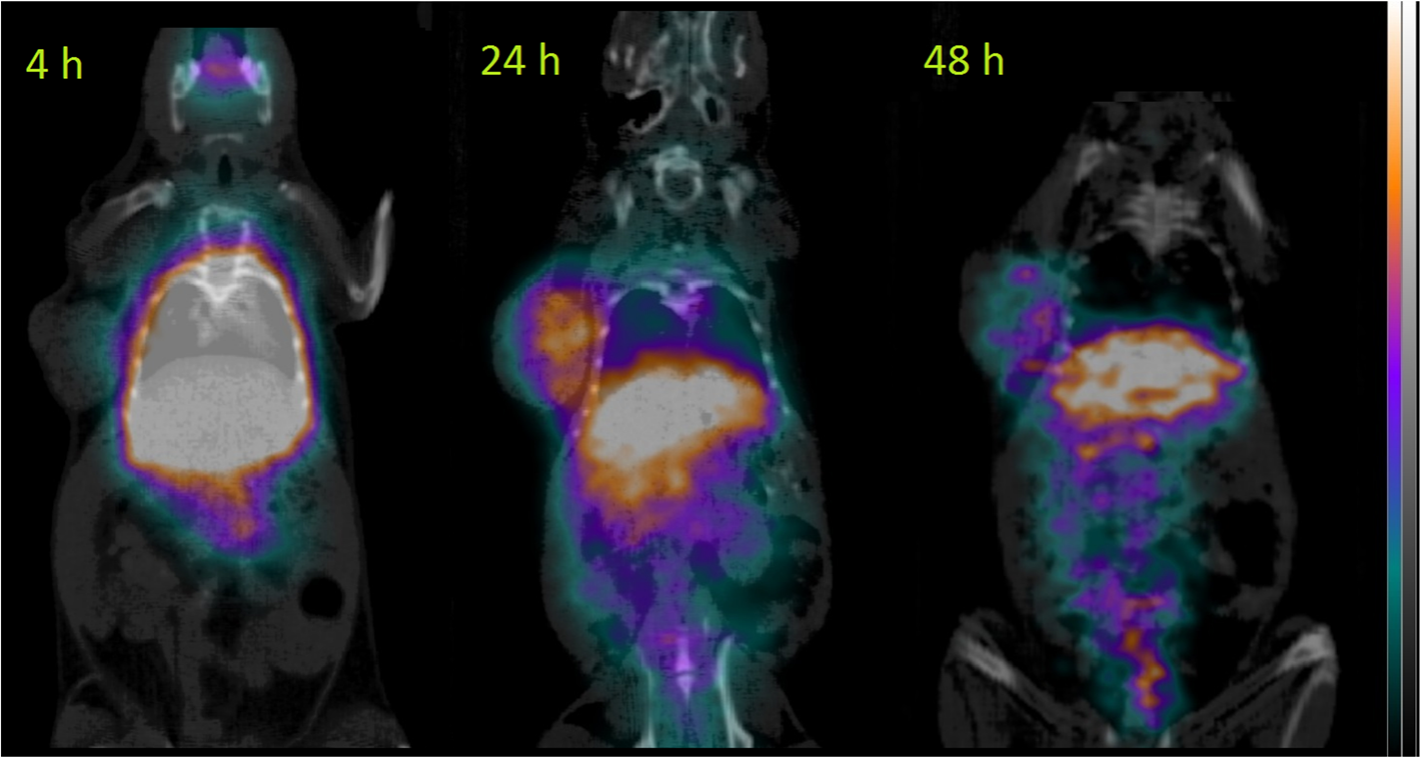
MicroPET longitudinal imaging of ^152^Tb-CHX-DTPA-scFv78-Fc targeting TEM-1 in a mouse bearing a subcutaneous RD-ES tumor

**Fig. 4:**
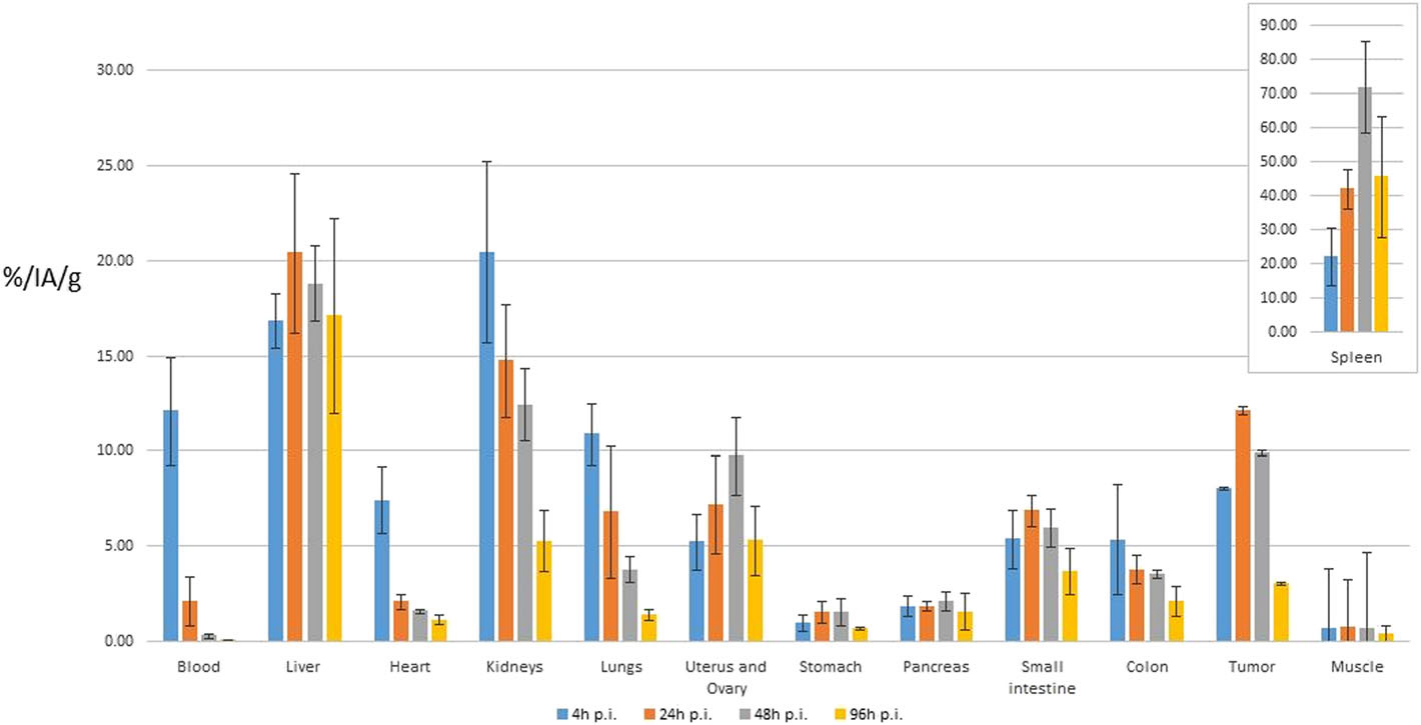
Biological organ kinetic of the radiolabeled antibody corrected for radioisotope physical decay. For each time point, color bars represent the average percent of injected activity per gram of tissue (%IA/g) ± 1SD

MicroPET images showed progressive clearance from the blood pool and increased tumor uptake between 4 h and 24 h post-injection. Most of the radioactivity was seen in the liver, with consequent apparent spill-out in adjacent thoracic and abdominal organs. The last time-point image showed persistent uptake in the abdomen and in the tumor, although with inferior image quality due to radioactive decay (Fig. 3). Complete decay-corrected pharmacokinetics, as resulted from ^111^In-CHX-DTPA-scFv78-Fc biodistribution experiments, is shown in Fig. 4. In the spleen and the female pelvic organs uterus and ovary, a constant biological uptake was seen up to 48 h after tracer injection, followed by washout 96 h post-injection. In the microPET images, these organs are not easily identifiable, which represents a limitation of the imaging method.

**Fig. 5:**
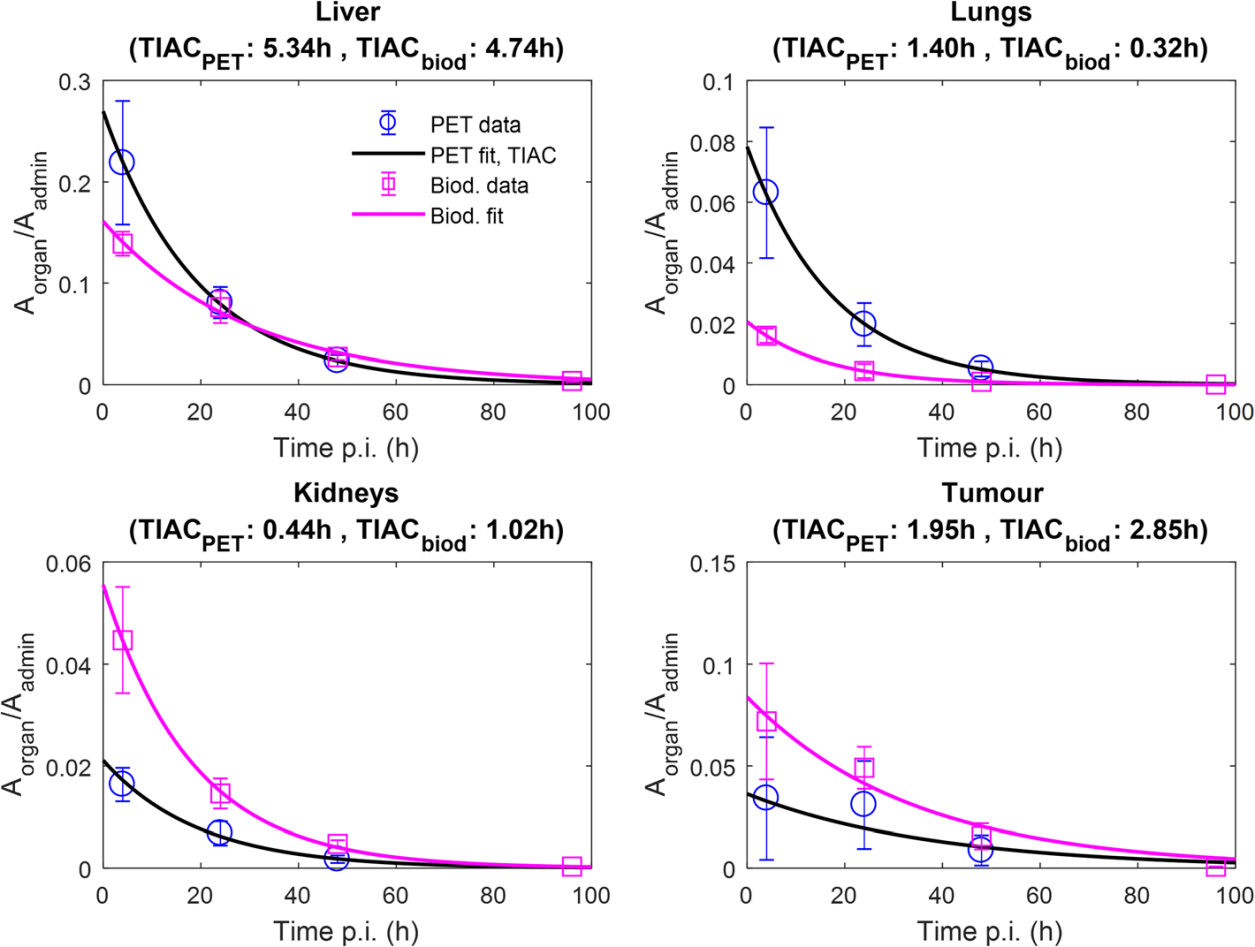
Normalized time-activity curves (nTAC) obtained for most relevant organs. The organ activity was normalized to the mice administered activity. Normalized activity time-points derived from PET are labeled with circles, while squares indicate data obtained from biodistribution experiments. Mean ± SD are reported for each time point. Full lines represent nTAC mono-exponential fits. The goodness of the fits was expressed by the *R*^*2*^ metric. *R*^*2*^ was > 0.96 for all fits calculated for the liver, lungs, and kidneys. *R*^*2*^ for the biodistribution-derived tumor nTAC fit was 0.97, whereas *R*^*2*^ for the imaging-derived tumor nTAC fit was 0.67

With the exception of the kidneys, all organ masses calculated by CT segmentation were larger than those calculated by harvesting and direct weighing of organs of similar-sized animals used for the biodistribution experiments. Specific organ and tumor masses calculated with the two methods are given in Table 1. Figure 5 shows the TIACs obtained with both modalities for most relevant compartments. Relative percent dose differences between microPET-based dosimetry and biodistribution-based dose extrapolations were + 12, − 14, and + 17 for the liver, the kidneys, and the tumors, respectively. Compared to extrapolations from biodistribution data, the imaging method significantly overestimated the absorbed doses to the heart and the lungs (+ 89 and + 117% dose difference, respectively). Full dosimetry results are given in Table 2.

**Table 1:** Organ and tumor masses used for dosimetry calculations. Mean organ and tumor masses obtained by direct harvesting and weighing, as well as those obtained by PET/CT imaging segmentation, are shown together with the standard organ masses implemented in the mouse model 25 g of OLINDA2. The standard organ masses of OLINDA2 were used for dose estimations in the organs that were not harvested or segmented in the biodistribution and microPET experiments, respectively (indicated with –)

Source organ	Average organ masses (g)		
	Biodistribution	MicroPET/CT	OLINDA2
Brain	–	–	0.46
LLI contents	0.76 (± 0.18)	1.19 (± 0.53)	0.58
Small intestine	1.26 (± 0.24)	3.56 (± 1.61)	1.74
Stomach	0.59 (± 0.20)	–	0.05
Heart wall	0.10 (± 0.01)	0.19 (± 0.08)	0.23
Kidneys	0.26 (± 0.03)	0.21 (± 0.11)	0.30
Liver	0.97 (± 0.15)	1.18 (± 0.26)	1.73
Lungs	0.17 (± 0.03)	0.24 (± 0.04)	0.08
Pancreas	0.10 (± 0.02)	–	0.30
Cortical bone	–	–	2.18
Spleen	0.04 (± 0.03)	–	0.11
Testes	–	–	0.16
Thyroid	–	–	0.01
Urinary bladder contents	–	–	0.06
Tumor	1.05 (± 0.35)	0.79 (± 0.20)	N.A.
Uterus and ovary	0.25 (± 0.07)	–	N.A.^†^

^†^A specific organ mass for mouse female reproductive organs is not available in the mouse model 25 g of OLINDA2

**Table 2:** Absorbed dose estimations of ^152^Tb-CHX-DTPA-scFv78-Fc in mice. The table reports dosimetry results obtained with the microPET-based method, as compared with the dose extrapolation from ^111^In-CHX-DTPA-scFv78-Fc biodistribution data. On the right, the relative percent difference between the two methods is given

Target organ	Absorbed doses (mGy/MBq)				
	microPET-based		Biodistribution-based		Relative difference (%)
	Mean dose	Std dev.	Mean dose	Std dev.	
Brain	6.34E + 01	2.84E + 01	3.94E + 01	1.27E + 01	60.98
Large intestine^†^	3.63E + 02	9.36E + 01	2.34E + 02	6.30E + 01	54.91
Small intestine^†^	3.45E + 02	8.84E + 01	3.29E + 02	6.00E + 01	4.71
Stomach wall	4.66E + 02	3.43E + 02	1.09E + 02	3.57E + 01	327.75
Heart wall^†^	6.28E + 02	1.41E + 02	3.31E + 02	7.70E + 01	89.65
Kidneys^†^	3.32E + 02	5.10E + 01	3.89E + 02	8.40E + 01	− 14.65
Liver^†^	5.90E + 02	1.27E + 02	5.24E + 02	9.70E + 01	12.60
Lungs^†^	3.20E + 02	4.58E + 01	1.47E + 02	4.00E + 01	117.86
Pancreas	2.24E + 02	2.56E + 01	3.60E + 02	7.50E + 01	− 37.78
Skeleton	1.11E + 02	2.27E + 01	6.57E + 01	1.81E + 01	68.99
Spleen	9.48E + 01	2.62E + 01	6.92E + 02	1.23E + 02	− 86.30
Testes	6.68E + 01	2.87E + 01	4.10E + 01	1.31E + 01	62.87
Thyroid	7.22E + 01	2.93E + 01	4.39E + 01	1.40E + 01	64.46
Urinary bladder	8.85E + 01	3.01E + 01	5.13E + 01	1.54E + 01	72.56
Total body	1.58E + 02	3.34E + 01	9.99E + 01	2.50E + 01	58.41
Tumor^†^	2.14E + 02	6.94E + 01	2.60E + 02	7.23E + 01	− 17.66

^†^Indicates organs that were segmented on microPET/CT imaging, for which the comparison between dose obtained with the two methods appears more meaningful. As regards dosimetry data to the heart, however, it should be noted that imaging-based data were obtained by manual contouring of both the heart wall and the heart content (see example of image segmentation in Fig. 2)

Results of the comparison between absorbed doses calculated with OLINDA2 and with the ellipsoidal model are reported in Table 3. OLINDA2, and the ellipsoidal model are in excellent agreement for spheres of masses > 1 g, where the average relative percent difference is − 2.9%. For spheres in the mass range 0.1–0.5, the difference increases to about − 10%.

**Table 3:** Dose factors for ^152^Tb uniformly distributed in spheres of unit-density tissue, according to OLINDA2 and to the ellipsoidal model described in [17–19]. Relative percent differences of OLINDA2 with respect to the ellipsoidal model are also reported

Sphere mass (g)	Sphere diameter (cm)	DFs (mGy/MBqh)		Relative difference (%)
		OLINDA2	Ellipsoidal model	
0.1	0.58	6.78E + 02	7.52E + 02	− 9.8
0.5	0.98	1.84E + 02	2.04E + 02	− 9.8
1	1.24	1.11E + 02	1.14E + 02	− 2.6
2	1.56	6.09E + 01	6.25E + 01	− 2.6
4	1.97	3.30E + 01	3.40E + 01	− 2.9
6	2.25	2.30E + 01	2.37E + 01	− 3.0
8	2.48	1.78E + 01	1.84E + 01	− 3.3
10	2.67	1.46E + 01	1.51E + 01	− 3.3

## Discussion

Sequential radionuclide imaging is becoming increasingly popular in preclinical studies as it allows the extraction of longitudinal data from single animals. For similar reasons, when studying dose/response predictions, imaging-based small animal dosimetry is theoretically advantageous over biodistribution-based dose extrapolations. In fact, if dosimetry data are extrapolated from living animals, the deterministic effects of radiation can be directly assessed, potentially driving a more accurate translation of therapeutic radiopharmaceuticals to the clinic. However, experience with imaging-based methods is still limited, and their validation against dose extrapolations from classical biodistribution experiments is necessary.

Bretin et al. compared microPET imaging and organ harvesting for mouse dosimetry of 6-[^18^F]fluoro-l-DOPA and 2-[^18^F]fluoro-l-tyrosine, showing a good correlation between the two methods [22]. However, in this study, the same set of reference organ masses was used, thus, the information on the inter-subject heterogeneity was lost and the technical differences between the two methods for absorbed dose estimations could not be exhaustively determined.

Denis-Bacelar et al. compared microSPECT imaging and organ harvesting for the calculation of %IA/g in subcutaneous tumors after the injection of ^111^In-labeled monoclonal antibodies [23]. They obtained a median %IA/g difference of about 30% between the two methods, resulting from discrepancies in the calculation of both tumor masses and activity concentrations. However, %IA/g was calculated from single time-points only, rather than from longitudinal imaging and biodistribution data [23].

In the present study, radiation dosimetry data were extrapolated from microPET sequential imaging of an antibody fragment labeled with the radio lanthanide ^152^Tb for the first time. This unconventional radionuclide was produced at the on-line isotope separator ISOLDE, at CERN, as part of the PSI-ISOLDE collaboration, and made available to research partners as a basis towards the frame of an international collaboration [24]. Due to its 17.5 h half-life, ^152^Tb appears suitable for imaging relatively long biological processes and, interestingly, it can be substituted with other radioisotopes of Tb (e.g., ^149^Tb, ^155^Tb, ^161^Tb) covering the entire spectrum of the medical applications of radionuclides [10]. On the other hand, ^152^Tb features a highly energetic positron and several prompt gamma emissions that may contribute to image degradation [11].

Our results show the feasibility of ^152^Tb microPET-based dosimetry with available hardware and software technology. In particular, for macroscopic subcutaneous tumors and organs at risk such as liver and kidneys, ^152^Tb microPET-based dosimetry compared well with dose extrapolations obtained from organ harvesting, with relative differences in the range 12–17%.

Notably, the biodistribution-based dosimetry was based on four data point (4 h, 24 h, 48 h, and 96 h), whereas the imaging-based dosimetry was based on three data points only (4 h, 24 h, 48 h). This was possible thanks to the longer half-life of ^111^In compared to ^152^Tb, and was functional to the objective of having a more robust gold standard against which to compare microPET-based dosimetry with ^152^Tb. However, the latest data point (96 h) had little impact on the extrapolated ^152^Tb-based organ TIACs. In fact, if the last measured data point was excluded, all organ ^152^Tb-based TIACs would have varied less than 6%, with the exception of a larger variation of 36% observed for the spleen (for example, the variation of the tumor TIAC was 4%, full data not shown). This is because 96 h following the injection (~ 6 effective half-lives), the remaining ^152^Tb activity is very low and gives only a little contribution to the determination of the TIAC. It may be argued that, for the biodistribution experiments, the use of ^152^Tb instead of ^111^In would have been optimal. However, this was not possible given the limited availability of ^152^Tb.

As expected, due to the significant spill-out activity from the liver in the living animal, image-based dosimetry of some organs, typically the lungs, suffers from a large overestimation compared to the biodistribution method, where organs are excised and counted separately. Nevertheless, these considerations might not hold true for radiopharmaceuticals that do not preferentially distribute in large abdominal organs such as the liver. The biodistribution pattern shown here is not due to specific TEM1 expression by the liver [25, 26], rather it is common to all antibodies bearing a functional Fc region [27].

Other organs, not specifically segmented on CT, show a general overestimation of the absorbed dose with the imaging-based method. This can be explained by the signal spill-out from the source organs that is consequently added to the remainder of the body. Radiation dose to the heart was also overestimated by the imaging method, due to its proximity to the liver and, probably, to the contribution of blood counts. In general, the presence of circulating blood might increase the signal in all living organs in PET-based dosimetry. In contrast, during organ harvesting, the blood is mostly washed out and does not contribute to the counting. However, this has probably only little effect in the present study, limited to the earliest time point, as the blood clearance of the scFv78-Fc was rapid (Fig. 4).

A major limitation of the imaging-based technique is the technical difficulty concerning the segmentation of small abdominal or pelvic organs. This is due to the small organ size which often cannot be resolved by the low-dose CT co-registered with small animal PET or SPECT. For example, in the present study, the biodistribution experiments revealed a prominent uptake in the spleen and in the female reproductive organs, which could not be translated into reliable corresponding image-based absorbed dose estimates because these body districts could not be adequately contoured on CT.

Another possible limitation of the study is the absence of partial volume effect (PVE) correction. Correction for PVE would have required a dedicated phantom study, which, in any case, could have not taken into account the signal blurring caused by the animal respiratory movements. Moreover, in the presence of organs with high uptake placed in close proximity, the validity of the application of PVE correction would have been questionable. On the other hand, PVE correction might have been useful for activity quantification in the tumors, which were far from the most active organs. However, as shown in Fig. 3, radioactivity was mainly contained within the tumor margins; therefore, the PVE effect was deemed minimal in this case.

Given that there are no previously available data on ^152^Tb dosimetry, we also compared the output of the spherical model of OLINDA2 with that of an independent analytical model for ellipsoidal shapes [17–19]. The two methods were highly concordant for masses > 1 g, while differences increased for smaller masses. The larger discrepancies for smaller masses could be attributable to the different calculation approaches for absorbed fractions and doses between the two methods [28], and might warrant further investigations.

## Conclusion

In this study, we demonstrated that microPET-based dosimetry of an unconventional radionuclide such as ^152^Tb is feasible, and the comparison with organ harvesting resulted in acceptable dose discrepancies for body parts that could be segmented on CT. These encouraging results warrant additional validation using radiolabeled molecules with a different biodistribution pattern.

## Data Availability

The datasets used and/or analyzed during the current study are available from the corresponding author on reasonable request.

## Abbreviations

%IA: percent of injected activity
CT: Computed tomography
DF: Dose factor
DTPA: Diethylenetriaminepentaacetic acid
Fc: Crystallizable fragment
FOV: Field of view
ICRP: International Commission on Radiological Protection
ISOLDE: Isotope mass Separator On-Line facility
iTLC: Instant thin layer chromatography
microPET: Small animal positron-emission tomography
microSPECT: Small animal single-photon emission computed tomography
nA: Normalized injected activity
NEMA: National Electrical Manufacturers Association
nTAC: Normalized time-activity curves
OLINDA2: OLINDA/EXM® 2.0
PVE: Partial volume effect
RADAR: Radiation Dose Assessment Resource
RD%: Relative percent difference
scFv: Single-chain variable fragment
SD: Standard deviation
TEM1: Tumor endothelial marker 1
TIAC: Time-integrated activity coefficient
VOI: Volume of interest
α-HIBA: α-hydroxyisobutyric acid
